# Assessing Whether Negative Parenting Cognitions Bias Parent Report of Preschoolers’ Externalizing Symptoms: A Regularized Moderated Non-Linear Factor Analysis Approach

**DOI:** 10.1007/s10802-024-01257-y

**Published:** 2024-11-15

**Authors:** Brigid Behrens, Katherine Edler, Kristin Valentino

**Affiliations:** Department of Psychology, University of Notre Dame, Notre Dame, IN 46556 USA

**Keywords:** Parenting cognitions, Externalizing symptoms, Regularized MNLFA, Preschool, Measurement

## Abstract

Parent report is frequently used to assess children’s psychopathology, however, researchers have expressed concerns about the validity of parent reports. Some parental characteristics, attitudes, or beliefs may systematically bias a parent’s report of their child’s behaviors and functioning. Informed by social information processing models of parenting risk, parents with more difficulties reflecting on and interpreting their children’s behavior may be at risk for less accurate reports. The present study conducted two regularized moderated non-linear factor analyses with LONGSCAN data to examine how parents’ self-reported negative parenting cognitions were associated with structural parameters of parent-reported child aggression and attention problems. While differential item functioning (DIF) was present on the aggression and attention problems subscales as a function of negative parenting cognitions, the DIF was small in magnitude, inconsistent in directionality and did not significantly alter factor-level parameters. Negative parenting cognitions did demonstrate a small but significant negative impact on all latent externalizing factors (aggression and attention problems), such that caregivers with fewer negative parenting cognitions endorsed fewer items, and this was associated with a lower mean of each latent variable. Given that accounting for DIF did not contribute to meaningful differences in impact parameters or improve criterion validity, findings suggest that the aggression and attention subscales are functionally invariant to negative parenting cognitions, suggesting that externalizing symptoms can be reliability compared across parents of varying parenting cognitions.

**Supplementary Information:**

The online version contains supplementary material available at 10.1007/s10802-024-01257-y.

Within research and clinical settings, parent reports are frequently used to assess children’s psychopathology. Parent report of the presence, severity, and impairment of children’s symptoms is time and cost efficient compared to labor-intensive assessment methods; it is also practical because young children have difficulties with reliable self-report, and parents spend a great deal of time observing their children’s behavior. In clinical settings, treatment often follows what the parent reports as problematic, even if the child disagrees (Hawley & Weisz, 2003). Despite the importance of parent report, researchers (e.g., Lau et al., 2006; Madsen et al., 2020; Olino et al., 2021; Richters & Pellegrini, 1989) have posited that certain parental characteristics, such as negative parenting cognitions, may bias parent report of child functioning (i.e., lead to systematic differences in the endorsements of items despite there being the same underlying level of the construct).

The importance of valid parent report is underscored by the long-lasting negative impacts of untreated psychopathology. Early externalizing symptoms (i.e., aggression, defiance) may have lingering effects into adolescence and adulthood as symptoms interfere with the successful achievement of developmental tasks that promote adaptive functioning (e.g., Finsaas et al., 2020; Sorcher et al., 2022). Thus, accurate parental identification of children’s externalizing symptoms is crucial. We used regularized moderated non-linear factor analysis to examine whether self-reported negative parenting cognitions—broadly defined as negative thoughts and beliefs about one’s own child and the caregiving role, and inaccurate knowledge about children and child development, and assessed in the present study as inaccurate developmental and family expectations, acceptance of physical discipline, and less empathy toward children—bias parents’ report of children’s externalizing symptoms among a sample at risk for negative parenting cognitions (i.e., parents at risk for or with maltreatment perpetration histories) and for child externalizing symptoms (i.e., children who have experienced or are at risk for maltreatment).

## Social Information Processing and Parent Report

Social information processing (SIP) models of parenting risk (Azar et al., 2008; Azar & Weinzierl, 2005; Crittenden, 1993; Milner, 1993) are useful for conceptualizing how parents’ cognitions—specifically, their attitudes and beliefs—may bias parent report. SIP models emphasize the role of schemas, or knowledge structures that help with organizing past information and perceiving/interpreting new information, in shaping parenting. Key schemas underlying parenting behaviors include parents’ attitudes and beliefs about caregiving, their child, and child development (Azar & Weinzierl, 2005). Parental schemas must be understood as continuous and in context. First, instead of a dichotomous divide between parents with certain cognitions and parents without, parents who have less accuracy and flexibility in their schemas may demonstrate more difficulties processing complex child-related information. Second, attending to context is critical, as systemic factors including poverty, racism, acculturation stress, and trauma exposure oppress parents with marginalized identities and may shape parents’ attitudes and beliefs. With both the continuous nature of schemas and the importance of context in mind, positive attitudes about caregiving and children are thought to help parents modulate their responses to difficult child behavior (e.g., viewing coloring on the walls as creative instead of malicious) and promote adaptive parenting (Azar et al., 2008; Camilo et al., 2019). In contrast, parents who hold more inaccurate or negative beliefs about child intentionality, compliance, and caregiver efficacy may be more likely to engage in harsh parenting practices (Azar et al., 2008, 2017; Camilo et al., 2021). For instance, strongly held inflexible or simplistic schemas (e.g., children *must* listen to their parents) are more likely to be violated, whereas flexible and complex schemas permit subtle situational information to be integrated into understandings of child behavior (e.g., my child slept poorly last night and that might be why they are being difficult; Azar et al., 2008).

Valid report is a product of several SIP-relevant cognitive stages: comprehension and interpretation of the question presented, recall of relevant information related to the question, and selection of a response (Tourangeau & Rasinski, 1988). Thus, parent report relies on a parent’s ability to accurately perceive and interpret the frequency, intensity, and typicality of their child’s emotional and behavioral states. As parents’ attitudes and beliefs about their caregiving, their child, and child development provide an interpretive framework for perceiving children’s behavior, negative parenting cognitions (including inaccurate, inflexible, and simplistic schemas) may interfere with accurate parent report of child functioning.

Negative parenting cognitions may lend themselves to biased reports in a few ways. Parents with more negative beliefs about their children may display attentional biases and selectively attend to cues or behaviors that are congruent with their expectations and beliefs. Furthermore, parents with higher levels of negative beliefs about their children or their caregiving efficacy may be more inclined to interpret their child’s ambiguous behavior as difficult or problematic. Given that observer informants are more likely to attribute behavior to dispositional and not contextual causes (De Los Reyes & Kazdin, 2005), negative parenting cognitions may increase risk for overly negative reports of child behavior. Finally, parents with more inaccurate or inflexible schemas may struggle to process complex child-related information, resulting in parents misinterpreting or altogether missing child cues. These schemas may make it difficult for parents to update their understanding of developmentally normative behavior as their child develops (e.g., playing loudly, difficultly sharing, frequency of tantrums), which could result in the perception of normative behavior as problematic or requiring intervention.

## Parent Report of Early Childhood Externalizing Problems

Understanding whether negative parenting cognitions influence the accuracy of parent report is especially crucial during early childhood (ages 4–6) because children in this age range are generally unable to provide reliable self-report (Measelle et al., 2005). Furthermore, during early childhood, children are not universally enrolled in out-of-home care, so there are few other informants who interact with children as frequently as their parents. While negative parenting cognitions are undoubtably important for parent report of all child psychopathology, it is important to examine parent report of child externalizing symptoms, which are one of the most common behavioral health complaints within early childhood (Ghandour et al., 2019). A parent with more negative schemas may have attentional biases toward observable misbehavior and/or a lower threshold for what they consider problematic, leading to endorsement of common externalizing problems such as aggressive behavior and attention problems. Indeed, informed by research on strained parent-child relationships (e.g., those with a maltreatment perpetration history), parents with a maltreatment perpetration history demonstrate more negative parenting cognitions (Stith, 2009), and endorse more behavioral problems in their children, but report similar levels of internalizing symptoms than parents without a maltreatment perpetration history (Lau et al., 2006). This pattern of results lends some support the interpretation that parents with more negative cognitions may be hyperreactive to and less tolerant of disruptive behaviors (Bauer & Twentyman, 1985; Lau et al., 2006). Altogether, negative parenting cognitions may reflect a transdiagnostic risk factor for biased parent reports of child psychopathology, particularly early childhood externalizing symptoms.

It is essential to examine whether negative parenting cognitions lead to over-reporting of children’s externalizing symptoms because of the clinical implications this phenomenon would have for diagnosis and treatment decision making. Excessive endorsement of child externalizing symptoms could reflect impaired family functioning, and it may be useful for such families to be connected with clinicians for assessment and intervention. However, accurate identification of specific symptoms and impairment is crucial because diagnosis and treatment follows from these assessments. If parents are either over- *or* under-reporting their children’s externalizing symptoms, children are less likely to receive the care that is most appropriate. Furthermore, early childhood aggressive behavior and attention problems may have long-lasting negative impacts (e.g., Finsaas et al., 2020; Sorcher et al., 2022) and some data suggest that parents of boys report more symptoms than parents of girls (Egger & Angold., 2006). Given these clinical implications, it is important to identify the extent to which negative parenting cognitions bias parent report of youth externalizing symptoms.

## Methods to Assess for Reporting Biases

Measurement invariance (MI) and differential item functioning (DIF) are two useful methods for evaluating reporting bias. MI assesses whether the relationship between observed indicators (e.g., an item about an externalizing symptom) and a latent construct (e.g., overall externalizing behavior) are the same, or invariant, across groups or time. MI is important for determining the validity of measurement across people with different characteristics (Bauer, 2017; van De Schoot et al., 2012). The absence of MI may result in a measure consistently over- or under-estimating a latent trait for a group, obscuring true mean differences by group (Millsap, 2011). A similar construct, DIF, emerges from the Item Response Theory literature (Osterlind & Everson, 2009). DIF occurs when the measurement properties of an item differ for one group versus another, regardless of true mean differences (Woods & Grimm, 2011). In other words, DIF identifies instances where people, who have the same level of the latent construct being measured, do not have the same likelihood of answering the item in the same way. Thus, both MI and DIF can identify whether negative parenting cognitions alter the ways in which parents interpret and respond to questionnaire items reflecting child externalizing symptoms.

MI and DIF are assessed using latent variable modeling approaches—multi-group MI or Multiple-Indicator Multiple-Cause (MIMIC) models, respectively. For multi-group MI, repeated confirmatory factor analyses (CFAs) with increasingly strict constraints are run (Chen, 2007; Horn & McArdle, 1992; Labouvie & Ruetsch, 1995). First, configural invariance examines if factor structure is the same across groups. Then, metric invariance tests if factor loadings are equivalent across groups. Third, scalar/threshold invariance constrains items intercepts to be the same across groups. Finally, strict/residual invariance assesses if random error is equivalent across groups. Despite the utility of multi-group MI, this method requires invariance to be tested as a function of categorical variables. Alternatively, DIF is commonly assessed with MIMIC models, which can accommodate multiple predictors, categorical *and* continuous. MIMIC models include direct paths from an independent predictor to a latent variable and the latent variable’s observed indicators. DIF is indicated if the path between a predictor and an item is significant while there is no relationship between the predictor and the latent variable. That is, an item is “easier” for some people, despite having the same level of the underlying construct (Montoya & Jeon, 2020). Still, MIMIC models assume equality of factor loadings and homogeneity of the variance-covariance matrix, which limit the assessment of some forms of DIF (Bauer, 2017).

Moderated non-linear factor analysis (MNLFA), and more recently regularized MNLFA, incorporates the strengths of MI and MIMIC models (Bauer, 2017), allowing for invariance to be tested across all parameters (factor means and variances, covariances, item intercepts, and factor loadings) as a function of multiple variables simultaneously, including continuous variables (Bauer, 2017; Belzak & Bauer, 2020; Bauer & Hussong, 2009). Specifically, MNLFA assesses MI/DIF via moderation, and allows predictors/covariates to be modeled as impact or DIF (Bauer, 2017). Impact represents true differences in the latent construct as a function of the predictor and is typically measured at the factor mean or variance level (Cole et al., 2022). In contrast, DIF—which can be uniform or non-uniform—represents predictor effects on individual item-level parameters and reflects measurement artifact (Bauer, 2017; Bauer et al., 2020; Cole et al., 2021). Uniform DIF is similar to a main effect and occurs when some people are more likely than others to endorse an item more despite there being no difference in the latent variable. Non-uniform DIF suggests that despite there being the same level of the latent variable, the relationship between the item and the latent variable (i.e., the factor loading) is moderated by the predictor.

To illustrate DIF and impact in practice, Gottfredson and colleagues (2019) conducted a study examining impact and DIF on adolescent report of substance use. They found evidence for mean impact for adolescents whose parents had lower education, meaning that students whose parents had less education demonstrated higher average levels of alcohol use. That is, they found evidence for true mean differences in their outcome as a function of a predictor (in this case, adolescent substance was influenced by parental education). In contrast, race/ethnicity demonstrated uniform/intercept DIF on two items, indicating that when the true underlying level of alcohol use was held constant (e.g., the latent variables mean was equivalent across groups), Black students were more likely than White students to endorse certain items. In other words, DIF reflects differences in item level responses that do not correspond to differences in the latent construct while impact refers to group level differences in the latent construct that do correspond to item-level differences. Together, MNLFA allows for the assessment of covariate differences in the severity of externalizing symptoms (mean impact), in item-level endorsements at the same level of externalizing symptom severity (intercept/uniform DIF), and to the extent that individual items are reflective of externalizing symptom severity (factor loading/non-uniform DIF).

## Present Study

The aim of this study was to use separate MNLFA models to examine whether negative parenting cognitions (inaccurate developmental and family expectations, acceptance of physical discipline, and less empathy toward children) were associated with the structural parameters of parent-reported child externalizing symptoms, specifically aggression and attention problems. We hypothesized that negative parenting cognitions would be associated with bias—specifically, that parents with more negative parenting cognitions would rate children’s aggression and attention problems systematically higher than parents with fewer negative parenting cognitions. Further, we expected that child sex and assessment site would demonstrate impact, as there may be true differences in child aggression and attention problems related to these variables that are not measurement artifact; as noted below, recruitment sites differed in the criteria by which they recruited participants.

## Method

### Participants

Data were drawn from the Age 4 timepoint (children were approximately four years old) of the Longitudinal Studies of Child Abuse and Neglect (LONGSCAN), a five-site consortium of parallel longitudinal studies initiated in 1991 that followed children who were born between 1986 and 1994 (see Runyan et al., 1998 for additional details). The five sites sampled children and their primary caregivers with varying levels of exposure to maltreatment and early life adversity. The East cohort recruited families from a low-income pediatrics clinic; a subset of this sample experienced risk at either the parent or child level. The Midwest cohort enrolled families who had been reported to Child Protective Services for suspected maltreatment and matched neighborhood controls. The South cohort included children identified as high risk by a state public health tracking database. The Northwest cohort included children at moderate risk for maltreatment who been referred to Child Protective Services prior to age 5. Finally, the Southwest cohort enrolled children who had entered an out-of-home placement due to confirmed maltreatment during their first 42 months of life. The term “parent” is used here for simplicity;more than 75% of caregivers were biological mothers.

Informed by SIP and cognitive behavioral theories of maltreatment, parents who have perpetrated maltreatment, especially physical abuse, tend to be, on average, high in negative parenting cognitions (Azar et al., 2008; Azar & Weinzierl, 2005; Milner, 1993; Stith et al., 2009). Moreover, children who have experienced child maltreatment are at heightened risk for externalizing symptoms (Kim-Spoon et al., 2013). Thus, this sample of parents who have perpetrated or are at risk for maltreatment was chosen given its likelihood of providing sufficient variation in negative parenting cognitions and child externalizing symptoms. However, it is essential to note that these processes operate in the context of broader systems and factors (e.g., poverty, racism, acculturation stress, trauma exposure) that may lead parents with low socioeconomic status and diverse racial and ethnic identities to be marginalized, involved in the child welfare system, experiencing their own psychopathology, and endorsing negative parenting cognitions.

Item-level missingness on the Child Behavior Checklist (CBCL 4–18; Achenbach, 1991) and Adult-Adolescent Parenting Inventory (AAPI, Bavolek, 1984) was less than 5% and thus, list-wise deletion was used to reduce computational overhead involved in multiple imputation (Schafer, 1999; Woods et al., 2021). Of the 1,129 families assessed at the Age 4 timepoint with completed data, this resulted in the following sample sizes: 1,118 for aggression and 1,123 for attention problems.

Demographic characteristics of the sample by site are presented in Table 1 by cohort. Across cohorts, children had a mean age of 4.3 (*SD* = 0.6), and approximately half (49%) were boys. The sample was racially and ethnically diverse with 52% of children identified as Black, 24.8% of children identified as White, 10% of children identified as Multi-Racial, 7.6% of children identified as Hispanic; 7.2% of children were missing racial and ethnic identify data. Finally, across all five sites, the sample was largely low-income; 62.1% of the sample reported having an income of 20,000 dollars or less, and the majority of families were on Medicaid (64.2%) and received food stamps (54.1%). Regarding other forms of public assistance, 21.4% of the sample endorsed receiving housing assistance and 31.9% endorsed receiving WIC.

**Table 1:** Sample descriptives

						Child Ethnicity						
Site	N	Child Age	Parent AAPI Mean (SD)	AAPI Range	Percent Male Child	White N (%)	Black N (%)	Hispanic N (%)	Multi-Racial N (%)	Other N (%)		Missing N (%)
Total Sample	1129	4.3 (0.6)	30.18 (4.64)	14–40	556 (49.24)	280 (24.8)	547 (52.0)	86 (7.6)	113 (10)	22 (0.02)	81 (7.2)	
Midwest	202	4.2 (0.49)	30.88 (4.09)	17–40	98 (48.5)	27 (13.37)	99 (49.00)	33 (16.34)	22 (10.90)	4 (0.02)	17 (8.4)	
South	203	5.27 (0.57)	27.83 (4.42)	14.75–38.25	94 (46.3)	70 (34.5)	111 (54.68)	0	1 (0.5)	0 (0)	21 (10.84)	
Northwest	216	4 (0)	32.28 (4.2)	19.75-40	109 (50.5)	98 (45.37)	44 (20.37)	5 (2.4)	46 (21.3)	11 (0.05)	12 (5.6)	
Southwest	294	4.16 (0.37)	31.37 (4.47)	18–40	137 (46.6)	75 (25.5)	96 (32.7)	47 (16.0)	42 (14.3)	7 (0.02)	29 (9.9)	
East	214	4 (0)	27.97 (4.16)	14-38.75	118 (55.1)	10 (4.7)	197 (92.1)	1 (0.4)	2 (0.9)	2 (0.01)	2 (0.9)	

*Note* AAPI = Adult-Adolescent Parenting Inventory (Bavolek, 1984)

### Ethical Considerations

Informed Consent was obtained from all participating families. Each LONGSCAN site and the coordinating center had IRB approval and a Federal Certificate of Confidentiality. More details on the consent and IRB process are available in Runyan et al. (1998).

### Measures

#### Negative Parenting Cognitions

Parent-report negative parenting cognitions were assessed with the 32-item Adult-Adolescent Parenting Inventory (AAPI, Bavolek, 1984). Items reflect whether parents have appropriate developmental expectations (e.g., “parents should expect children to feed themselves by 12 months”), empathy toward children (e.g., “young children who are hugged and kissed will grow up to become ‘sissies’”), rejection of physical discipline (e.g., “children should be forced to respect parental authority”), and appropriate family roles (e.g., “young children should be expected to comfort their mother when she is feeling blue”). We created an AAPI sum score, which had strong internal consistency (α = 0.94). Items were rated on a 5-point scale ranging from 1: strongly agree to 5: strongly disagree, such that lower scores indicated *more* negative parenting cognitions.

#### Child Externalizing Symptoms

Child externalizing symptoms was assessed with the 113-item, caregiver-report CBCL 4–18 (Achenbach, 1991). Caregivers completed the CBCL at the age 4 visit date, responding to questions their child’s emotional and behavioral problems on a three-point ordinal scale of 0 to 2 (0: Not True, 1: Somewhat or Sometimes True, 2: Very or Often True). Generally, the CBCL produces eight syndrome scales: Social Withdrawal, Somatic Complaints, Anxiety/Depression, Social Problems, Thought Problems, Attention Problems, Delinquent Behavior, and Aggressive Behavior. In addition, research on the factor structure of the CBCL has suggested that syndrome scales can be organized into composites for Externalizing and Internalizing symptoms (Achenbach, 1991; Greenbaum & Dedrick, 1998). In the present study, we examined one of the syndrome scales that are used for Externalizing composite score creation—Aggressive Behavior—as well as the Attention Problems syndrome scale. There are 19 items on the Aggressive Behavior subscale and 10 items on the Attention Problems subscale. The Attention Problems subscale is not formally included in either composite score as it loads on both the Internalizing and Externalizing factor (Greenbaum & Dedrick, 1998), but was included in these analyses given the relationship between attention problems and externalizing symptoms. Finally, we did not include the Delinquent Behavior subscale due to infrequent item endorsement and low internal consistency (α_delinquency_ = 0.61).

The Aggressive Behavior and the Attention Problems subscale items were examined in terms of skew and kurtosis and response frequencies (see Table 1 in the Supplemental Materials). There was substantial positive skew across items. Thus, items that had sparse upper distributions (operationalized as less than 10% of the sample endorsing a response option) were dichotomized to indicate the presence of a symptom (0 = no, 1 = yes). This resulted in 10 items on the Aggression Behavior subscale (items: 7, 16, 20, 21, 22, 37, 57, 87, 94, 97) and 8 items on the Attention Problems subscale (items: 1, 13, 17, 41, 45, 46, 62, 80) being dichotomized (see Table 2 in the Supplemental Materials). This is an approach that assists in model convergence when the frequency of responses is low for certain responses on a scale (Connell et al., 2021; Curran et al., 2016). In addition, several CBCL items were inappropriate or irrelevant for four-year-olds; these items had high rates of missingness and/or showed little to no variance. Given this, two items (item 23 from the Aggressive Behavior subscale and item 61 from the Attention Problems subscale) were excluded. Cronbach’s alpha and average inter-item correlations demonstrated adequate to strong internal consistency for these modified subscales (α_aggression_ = 0.88; α_attention_ = 0.75).

**Table 2:** Latent means and variances of the aggression subscale: using scoring models that do and do not account for differential item functioning

	DIF-Adjusted						Unadjusted					
	Mean Impact			Variance Impact			Mean Impact			Variance Impact		
Covariate	Est.	S.E.	p	Est.	S.E.	p	Est.	S.E.	p	Est.	S.E.	p
Male	0.030	0.033	0.543	0.139*	**0.055**	**0.018**	0.041	0.033	0.272	0.149**	**0.055**	**0.014**
AAPI	− 0.069	0.036	0.098	0.056	0.053	0.404	− 0.060*	**0.035**	**0.040**	0.084	0.054	0.515
Site: South	− 0.046	0.041	0.397	0.021	0.069	0.812	− 0.046	0.042	0.219	0.041	0.069	0.909
Site: Northwest	0.142**	**0.041**	**0.001**	− 0.032	0.068	0.553	0.142**	**0.041**	**0.001**	− 0.034	0.068	0.575
Site: Southwest	0.061	0.040	0.110	0.036	0.065	0.530	0.055	0.040	0.113	0.019	0.065	0.536
Site: East	− 0.061	0.043	0.151	0.056	0.070	0.378	− 0.059.	0.043	0.157	0.060	0.070	0.415

*Note* DIF = Differential Item Functioning; AAPI = Adult-Adolescent Parenting Inventory. The Male variable is effects coded with female as the reference group. Site is effects coded with the Midwest sample being the reference group. Because factor level effects are considered latent differences in the construct, in regularized MNLFA, the penalty function is not applied to factor level effects. Thus, p values are interpretable. *p <.05, **p <.01,

#### Consulted about Difficulties

For an exploratory criterion validity analysis, one additional measure from the Age 6 timepoint was included. Parent consultation due to child behavioral problems was assessed using a single item yes/no question that asked parents, “during the past year, have you consulted with anyone about a behavioral, emotional, or school problem related to (child name)?” A response of 1 indicated that the parent had consulted with someone, a response of 0 indicated that the parent had not consulted with someone.

### Analytic Plan

Two regularized MNLFAs (Bauer et al., 2020; Belzak, 2023) using least absolute shrinkage and selection operator (LASSO; Tibshirani, 1996) were conducted using regDIF in R (Belzak, 2021, 2023), one for the modified Aggressive Behavior subscale (“Aggression”) and one for the modified Attention Problems subscale (“Attention”). As described in the introduction, MNLFA generalizes traditional latent variable models to include the simultaneous assessment of MI/DIF as a function of multiple variables; therefore, in each MNLFA, child sex (effects coded with female as the reference group), site (effects coded with the Midwest group as the reference group), and negative parenting cognitions were all assessed for impact and DIF. Child sex was included as a predictor because of past research has been shown to be associated with differences in externalizing symptoms (Egger & Angold., 2006). Assessment site was included as a predictor because, as noted previously, the LONGSCAN dataset included five cohorts/sites that each had different recruitment criteria which may have led to differences in our primary study variables; for example, while the East cohort recruited families from a low-income pediatrics clinic, the Southwest cohort recruited families with children who had entered an out-of-home placement due to confirmed maltreatment during their first 42 months of life.

The ability of MNLFA to simultaneously assess MI/DIF as a function of multiple variables comes at a cost of model complexity; the number of possible model comparisons and ways that DIF may manifest increases dramatically (Bauer et al., 2020; Belzak & Bauer, 2020). In response to these challenges, Bauer and colleagues (2020) proposed applying regularization techniques, such as LASSO. Essentially, regularization leads to model parsimony by adding in a cost for each parameter added, where DIF effects that do not meaningfully improve the model are forced to zero. A tuning parameter (also called a penalty parameter) controls the strength of the penalty term; as the tuning parameter is reduced, more DIF is allowed into the model until too many DIF effects are included and the model becomes unidentified. The goal of regularization is to identify the optimal penalty term (Belzak, 2023). The best fitting model minimizes the Bayesian Information Criterion (BIC) value, and the presence of DIF is determined by which of the items have non-zero DIF effects (Bauer et al., 2020; Belzak & Bauer, 2020). Importantly, the tuning parameter is only applied to DIF, and baseline and impact parameters are not penalized. Studies have found that regularized MNLFA (using a LASSO penalty) demonstrated a better balance of Type 1 and Type 2 errors compared to other methods of DIF assessment (Bauer et al., 2020; Cole et al., 2022). In addition, the use of regularization avoids the need for item-by-item, sequential testing, and the need to specify a priori anchor items. Specifically, in unpenalized MNLFA, sequential models are fit where DIF is permitted on one item at a time and all remaining items are treated as invariant anchors (e.g., no DIF) (Bauer et al., 2020). Regularized MNLFA does not require any a priori anchor items as, dependent on the penalty term, some of the DIF parameters will be shrunk to zero. Finally, regularized MNLFA is automated, reducing computational overhead (Belzak, 2023).

For these reasons, regularized MNLFAs using LASSO were conducted to evaluate our hypotheses; as noted, child sex, site, and negative parenting cognitions were assessed for impact and DIF in each regularized MNLFA. Separately for each subscale, a baseline model was first run that did not allow for DIF but estimated impact on the latent construct mean and variance (the unadjusted model). Then, following other applications of regularization to latent variable models (Jacobucci et al., 2016), BIC values are produced for 100 possible values of the tuning parameter, starting with the most restrictive model (i.e., a large penalty term, no DIF included). The best fitting model and the optimal tuning parameter were determined by lowest BIC value (Belzak & Bauer, 2020). In the final models, we examined any significant impact and DIF. Finally, latent factor scores were calculated using Expected a Posteriori estimation for both the unadjusted model and the DIF-adjusted model. In addition, the unweighted simple sum score was calculated. Correlations were run among the unadjusted score, the DIF-adjusted score, and the unweighted sum score.

## Results

### Configural Invariance

As MNLFA analyses assume configural invariance, configural invariance analyses were also conducted on each subscale (Aggression and Attention), as a function of high and low negative parenting cognitions (using a median split). Using a median split as the grouping variable, the models indicated configural invariance.

### MNLFA for Aggression

The 24th model was the best fitting (BIC = 30,393.41; tuning parameter = 0.21); it allowed DIF on 12 parameters and constrained other DIF parameters to 0. There were some uniform DIF effects by site and child sex, indicating that some parents were more likely than others to endorse an item more despite there being no difference in the latent variable (see Fig. 1 in the Supplemental Materials). Parents in the East vs. Midwest cohorts (estimate = − 0.04) and the South vs. Midwest cohorts (estimate = − 0.06) were less likely to endorse item 16 (bullies others). Parents in the South vs. Midwest were also less likely to endorse item 21 (destroys other people’s items; estimate = − 0.07). Parents in the East vs. Midwest cohorts (estimate = 0.01) and parents of male children (estimate = 0.26) were more likely to endorse item 20 (destroys items). Parents of male children were also more likely to endorse item 97 (threatens others; estimate = 0.11).

There was uniform DIF as a function of negative parenting cognitions on four items. At similar levels of the aggression factor, parents with fewer negative parenting cognitions were more likely to endorse item 22 (noncompliant at home; estimate = 0.26), and less likely to endorse items 27 (jealous, estimate = − 0.07), 94 (teases; estimate = − 0.07), and 104 (very loud, estimate = − 0.16); thus, negative parenting cognitions both increased and decreased the threshold at which parents endorsed items. Non-uniform DIF as a function of negative parenting cognitions was observed for two items, indicating that despite there being the same level of the latent variable, the relationship between the item and the latent variable was moderated by the predictor. These were item 57 (physically hurts others, estimate = 0.12) and item 97 (threatens others, estimate = 0.12). These items were therefore more discriminating and central to the aggression factor for parents with fewer negative parenting cognitions.

The correlation between the DIF-adjusted and unadjusted factor scores for aggression was very high (*r* =.99), as were the correlations between the unweighted sum score and the DIF-adjusted and unadjusted factor scores (*r*s = 0.98). Mean and variance impact parameters for the unadjusted and DIF-adjusted model are presented in Table 2. Examining the differences in factor means and variances in the DIF-adjusted versus unadjusted models, the effects of accounting for DIF on the mean/variance parameters was minimal. Thus, accounting for DIF did not contribute to meaningful differences in the mean, variance, or factor score of the aggression subscale, so the unadjusted model is interpreted for impact. Parents of children in the Northwest vs. Midwest cohort had higher item responses as a function of higher scores on the latent aggression variable. Parents with fewer negative parenting cognitions endorsed significantly fewer items as a function of lower scores on the latent aggression variable. Finally, compared to caregivers of girls, parents of boys demonstrated more variability in their report of aggression.

### MNLFA for Attention Problems

The 11th model was the best fitting (BIC = 13,648.17, tuning parameter = 0.27); it allowed for one DIF parameter and constrained all other DIF parameters to 0. One uniform DIF effect emerged on Item 41 (child acts impulsively) as a function of negative parenting cognitions (see Fig. 2 in the Supplemental Materials). Parents with fewer negative parenting cognitions were more likely to endorse that their child was impulsive (DIF estimate = 0.12).

There was a very high correlation (*r* =.99) between the DIF-adjusted and unadjusted factor scores for attention problems, and between the unweighted sum score and the DIF-adjusted and unadjusted factor scores (all *r*s = 0.94). When examining the differences in factor means and variances in the DIF-adjusted versus unadjusted models, the effects of accounting for DIF on the mean/variance parameters was minimal; therefore, the unadjusted model is interpreted for impact. Mean and variance impact parameters for the unadjusted and DIF-adjusted model are presented in Table 3. As compared to the Midwestern cohort, parents of children in the South, Northwest and Southwest cohorts had higher item responses as a function of higher scores on the latent attention problems variable; those in the East cohort displayed lower item responses as a function of lower scores on the latent attention problems variable. In addition, parents of boys endorsed significantly more items than parents of girls as a function of higher scores on the latent attention problems variable. Parents with fewer negative parenting cognitions endorsed significantly fewer items as a function of lower scores on the latent attention problems variable. Finally, parents of children from the South vs. Midwest cohort demonstrated less variability in their report of attention problems.

**Table 3:** Latent means and variances of the attention problems subscale: using scoring models that do and do not account for differential item functioning

	DIF-Adjusted						Unadjusted					
	Mean Impact			Variance Impact			Mean Impact			Variance Impact		
Covariate	Est.	S.E.	p	Est.	S.E.	p	Est.	S.E.	p	Est.	S.E.	p
Male	0.115**	**0.037**	**0.002**	− 0.012	0.071	0.872	0.115**	**0.037**	**0.002**	− 0.011	0.072	0.882
AAPI	− 0.119**	**0.040**	**< 0.001**	0.107	0.068	0.113	− 0.107**	**0.009**	**0.007**	0.086	0.068	0.211
Site: South	0.132**	**0.042**	**0.002**	− 0.213*	**0.097**	**0.028**	0.132**	**0.071**	**0.002**	− 0.208*	**0.097**	**0.031**
Site: Northwest	0.149**	**0.045**	**0.001**	− 0.100	0.089	0.258	0.149**	**0.075**	**0.001**	− 0.103	0.089	0.247
Site: Southwest	0.187**	**0.046**	**< 0.001**	0.083	0.084	0.322	0.187**	**0.07**	**< 0.001**	0.080	0.084	0.340
Site: East	− 0.257**	**0.052**	**<0.001**	0.141	0.089	0.113	− 0.258**	**0.086**	**< 0.001**	0.140	0.089	0.114

*Note* DIF = Differential Item Functioning; AAPI = Adult-Adolescent Parenting Inventory. The Male variable is effects coded with female as the reference group. Site is effects coded with the Midwest sample being the reference group. Because factor level effects are considered latent differences in the construct, in regularized MNLFA, the penalty function is not applied to factor level effects. Thus, p values are interpretable. * *p* <.05, ** *p* <.01, *** *p* <.001

### Exploratory Criterion Validity Analysis

To further examine the utility of the different scoring methods and to gain insight into the practical implications of accounting for DIF, correlations were compared between the three different scoring methods (unadjusted, DIF-adjusted, and traditional/unweighted scores) and one variable assessed approximately two years later that we hypothesized would be related to child externalizing symptoms; caregiver endorsement of consulting with someone about their child’s behavioral, emotional, or school problems. For aggression and attention problems, all three scoring methods (unadjusted, DIF-adjusted, and unweighted sum scores) demonstrated a modest, significant, positive correlation with endorsement of consulting with another about their child’s difficulties two years later (*r*_*aggression*_ = 0.16 − 0.17; *r*_*attention*_ = 0.21 − 0.22). While the unadjusted factor scores demonstrated the highest correlations with the six-year variable, the magnitude of this improvement was minimal to modest. This suggests that accounting for DIF when creating factor scores did not have any meaningful advantages over other, less complex scoring methods (e.g., unadjusted factor scores or sum scores).

## Discussion

Parent report of child functioning is a cornerstone of psychological research and clinical services, but there are concerns about parent characteristics that might systematically bias parent report (e.g., Madsen et al., 2020; Olino et al., 2021; Richters & Pellegrini, 1989). We examined whether negative parenting cognitions were associated with parent-reported child externalizing symptoms. Minimal DIF (related to negative parenting cognitions) was found for child attention problems, and there was some DIF for child aggression related to negative parenting cognitions, site, and child sex. Negative parenting cognition-DIF did not consistently occur in one direction; for parents of children with equivalent levels of total aggression or attention problems symptoms, negative parenting cognitions were associated increased endorsement of some items but decreased endorsement of others. To better understand the implications of this DIF, factor scores were compared, and a criterion validity analysis was conducted. Because accounting for DIF did not contribute to meaningful differences in factor-level parameters or markedly improve criterion validity, the hypothesis that negative parenting cognitions would bias parent report of child externalizing symptoms was not supported.

### Bias and Negative Parenting Cognitions

Interestingly, despite some evidence for item-level non-invariance as a function of negative parenting cognitions on the aggression and attention problems subscales, accounting for this DIF did not result in meaningful changes in the latent parameters of these subscales. This may have been because the direction of DIF related to negative parenting cognitions was inconsistent and the DIF was limited and small to moderate in magnitude. Because accounting for DIF did not markedly alter factor-level parameters or factor scores, all subscales were functionally invariant and unique DIF pathways are not interpreted (Cole et al., 2022).

These findings add to the small but growing body of literature using psychometric methods to assess in bias in parent-reported measures (Olino et al., 2021; Rancourt et al., 2022; Stevens et al., 2022). We found that parents’ ratings of their children’s externalizing symptoms were not systematically and meaningfully related to negative parenting cognitions, and therefore not a source of bias. These results mirror findings from Olino et al. (2021), who used MNLFA to assess bias due to maternal psychopathology symptoms and found limited to no evidence for bias. Taken together, these findings indicate that parent report may be more robust to the presence of negative parenting cognitions and psychopathology than previously thought. Still, these findings contrast prior literature, and the depression-distortion hypothesis (Richters & Pellingrini, 1989), which argue that certain parental characteristics associated with depression interfere with the ability to report on child symptoms (e.g., Fergusson et al., 1993; Madsen et al., 2020; Richters & Pellegrini, 1989). As noted previously, much of the parental bias literature has used methods such as informant-discrepancies that do not necessarily measure bias. Indeed, measuring bias as the disagreement between two informants is incompatible with the view that discrepant reports provide complementary information (De Los Reyes et al., 2023). In addition, parental depression is not analogous to negative parenting cognitions—depressive episodes and associated negative emotionality and cognitive biases vary notably across individuals and over episodes, while parenting cognitions likely develop over time as a product of dynamic factors at the child, parent, family, and community level (Azar et al., 2008).

Negative parenting cognitions also demonstrated a small but significant negative impact on all latent externalizing factors (aggression and attention problems). Caregivers with fewer negative parenting cognitions endorsed fewer items, and this was associated with a lower mean of each latent variable, suggesting true differences in the latent mean rather than bias. This finding builds upon the extensive body of research on bidirectional relations between parent and child functioning and coercive parent-child cycles (Cole et al., 2003; Patterson, 2002). Indeed, the significant relation between negative parenting cognitions and more child behavioral challenges may reflect a pattern wherein children receive attention for their difficult behaviors, inconsistent discipline, and insufficient or inconsistent parent attention for positive behaviors. These parenting responses may reinforce children’s behaviors and, over time, lead to the child engaging in more externalizing behaviors (Patterson, 2002).

While our findings broadly support coercive cycles, they might also speak to the importance of explicitly addressing parent cognitions in interventions for child externalizing behaviors, like behavioral parent training (BPT). BPT is an efficacious treatment for child externalizing concerns that focuses on enhancing parenting skills through increasing praise and parental consistency, positive reinforcement, natural consequences and time-out. Despite the considerable evidence for the efficacy of BPT, effectiveness varies and differs significantly based on several family variables including family size, income level, education, and single parent status (Reyno & McGrath, 2006). Given this gap between efficacy and effectiveness, theoretical and applied research (Azar et al., 2008; Chacko et al., 2016; Mah & Johnston, 2008; Novick et al., 2022) have suggested that explicitly addressing parenting cognitions could enhance the effectiveness of BPT or other parenting programs as cognitions may not only predict parenting behaviors, but may also influence how parents accept and engage with the content of BPT. Indeed, our finding that negative parenting cognitions were associated with more externalizing symptoms may provide further support for studying the benefits of including non-behavioral components (e.g., discussions about parenting beliefs, attributions and attitudes) into traditional BPT to enhance effectiveness.

### Site and Child Sex Factor-Level Effects

Across models, effects emerged as a function of site and child sex. The true differences due to site are not surprising, as sites intentionally sampled children of various risk levels. Being from the Northwest cohort (vs. the Midwest) was most consistently related higher scores on the aggression and attention problems latent variables. In contrast to the Midwest cohort, which included infants with and without maltreatment histories at enrollment, all children from the Northwest cohort had a recent referral to Child Protective Services and moderate risk for child maltreatment based on a state risk assessment system. While the Southwest cohort also represented a higher-risk sample (children were recruited due to a placement in foster care during their first 42 months of life), this site only demonstrated mean impact for attention problems. Recency of welfare involvement may contribute to these different externalizing profiles.

There was inconsistent support for child sex differences in parent-reported externalizing symptoms. Caregivers of boys reported significantly more attention problems and had significantly more variability in their reports of their aggression problems than caregivers of girls. This pattern lends support to research which suggesting sex differences in early childhood psychopathology (Egger & Angold., 2006). One possibility for this finding is that there is a reciprocal relationship between child gender and parenting style, such that gender influences the type or level of discipline, and this in term influences the expression of behaviors. However, more research should be done to better understand different profiles of externalizing symptoms in young boys and girls.

### Strengths and Limitations

A key strength of the present study was the use of regularized MNLFA to concurrently examine impact and DIF as a function of continuous and categorical predictors. The use of a regularization technique removed the need for sequential inference testing, did not require items to be specified as invariant anchors, and provided a better balance between Type 1 and Type 2 errors (Bauer et al., 2020). An additional strength is the use of a large, diverse sample at risk for negative parenting cognitions and externalizing symptoms.

Still, there are several limitations. Due to model complexity and computational burden, only unidimensional models were run. This limited our ability to create an overall, multi-factor externalizing symptoms score, or to include other commonly comorbid symptom domains. Additionally, results may have differed if another MNLFA method was used (e.g., aMNLFA). A study by Cole et al. (2022) examining the implications of different MNLFA methods found that while factor scores appeared relatively robust to different analytical decisions, DIF parameters were more sensitive. Thus, the present study’s findings should be replicated and the implications of different analytical decisions (e.g., which variables were dichotomized) on DIF estimates should be explored.

A second limitation is that, while the AAPI is a widely used and measure of negative parenting cognitions, it specifically captures inaccurate developmental and family expectations, acceptance of physical discipline, and less empathy toward children. It will be important for future work to examine potential DIF as a function of other SIP domains (i.e., parents’ thoughts and feelings about their child, parents’ attributions for their child’s behaviors, and their broader thoughts about the role of a caregiver). Researchers could consider using alternative measures such as the Parent Cognition Scale (Snarr et al., 2009) and the Parent Attribution Test (Bugental et al., 1989). Additionally, it would be interesting to see if similar results emerges if a more implicit measure of parenting cognitions is used; researchers could code five-minute speech samples with the Family Affective Rating Scale (Bullock & Dishion, 2007).

Third, the item we used to assess criterion validity was broad, reflecting whether parents had consulted with anyone about their child experiencing behavioral, emotional, or school problems. It is possible that parents may have endorsed this item due to other difficulties children are experiencing beyond just externalizing problems. Our examination of this item was supplemental and exploratory, so in future work, researchers might consider using alternative measures to establish criterion validity.

Finally, the present study was interested in the role that negative parenting cognitions had on measurement parameters, but the presence of such cognitions may depend on environmental context, which was not measured. For example, external stressors are associated with less positive beliefs about children (Conger et al., 1984), and so it may be that responses on the AAPI reflect, in part, factors such as neighborhood context, racism, and economic hardship. In addition, some items on the AAPI might be capturing culturally bound parenting beliefs or practices that have been adaptive to a family’s context. Thus, it is important to expand beyond internal parental characteristics and consider additional child, family-level, or contextual variables (e.g., child comorbidity, family stress, economic hardship, or racism), and the interactions between these variables. For instance, research has demonstrated that children of minoritized identities, especially Black children, have their emotions and behaviors more intensely monitored by authority figures (Halberstadt et al., 2018). Therefore, for families of minoritized racial and ethnic backgrounds, AAPI items including “children develop good strong character through very strict discipline” and “children quit crying faster when they are ignored” may be picking up on parenting practices employed to prepare children for systemic inequities, racism, and discrimination (Dunbar et al., 2017, 2021; Nelson et al., 2012). In future research, it will be important to examine how systemic factors such as racism and poverty are relevant for understanding the occurrence of reporting biases and, more broadly, negative parenting cognitions and child externalizing behaviors.

## Conclusions

In summary, the application of regularized MNLFA allowed for a comprehensive assessment of whether negative parenting cognitions bias parental responses to questions about their child’s externalizing symptoms. Although some prior research has identified discrepancies in parent report, interpreted these discrepancies as bias, and expressed concerns about the validity of parent report (e.g., Bradley & Peters, 1991; Fergusson et al., 1993; Lau et al., 2006; Madsen et al., 2020), we found limited evidence for consistent and meaningful negative parenting cognition-related DIF on parent-reported child externalizing symptoms. Indeed, the identification of DIF does not necessarily mean that the DIF is meaningful, that scoring needs to be changed, or that a report is invalid. Thus, findings reiterate the importance of examining the practical significance of any identified bias and provide evidence for the validity of parent-reported child externalizing problems. Given the extensive use of the CBCL in both clinical and research settings, this finding is promising and suggests that differences in child externalizing symptoms can be considered with parents endorsing varying levels of negative parenting cognitions.

## Electronic Supplementary Material

Below is the link to the electronic supplementary material.

Supplementary Material 1

## Data Availability

The data used were made available by the National Data Archive on Child Abuse and Neglect, Cornell University, Ithaca, NY, were used with permission.
