# Supplementary material for: Assessing Whether Negative Parenting Cognitions Bias Parent Report of Preschoolers’ Externalizing Symptoms: A Regularized Moderated Non-Linear Factor Analysis Approach
Source: Res Child Adolesc Psychopathol. 2024 Nov 15;53(2):207–20. doi: 10.1007/s10802-024-01257-y (PMC11845539; doi:10.1007/s10802-024-01257-y)
Supplement: Supplementary file 1 — Supplementary Material 1 [file 10802_2024_1257_MOESM1_ESM.docx]

**Supplemental Materials**

**Table 1**

*Item Frequencies for Externalizing Subscales*

| Scale | Item | M (SD) | Range | Skew |  | Kurtosis |  |
| --- | --- | --- | --- | --- | --- | --- | --- |
| Aggression | 3 | .94 (.75) | 0-2 | 0.09 |  | -1.2 |  |
| Aggression | 7 | .48 (.65) | 0-2 | 1.03 |  | -0.11 |  |
| Aggression | 16 | .35 (.56) | 0-2 | 1.34 |  | 0.82 |  |
| Aggression | 19 | .93 (.76) | 0-2 | 0.11 |  | -1.26 |  |
| Aggression | 20 | .43 (.64) | 0-2 | 1.22 |  | 0.29 |  |
| Aggression | 21 | .39 (.62) | 0-2 | 1.33 |  | 0.63 |  |
| Aggression | 22 | .67 (.62) | 0-2 | 0.37 |  | -0.68 |  |
| Aggression | 27 | .84 (.76) | 0-2 | 0.27 |  | -1.21 |  |
| Aggression | 37 | .25 (.52) | 0-2 | 1.94 |  | 2.87 |  |
| Aggression | 57 | .23 (.49) | 0-2 | 2 |  | 3.22 |  |
| Aggression | 68 | .47 (.67) | 0-2 | 1.1 |  | -0.05 |  |
| Aggression | 74 | .78 (.67) | 0-2 | 0.29 |  | -0.79 |  |
| Aggression | 86 | .85 (.67) | 0-2 | 0.19 |  | -0.82 |  |
| Aggression | 87 | .44 (.06) | 0-2 | 1.03 |  | 0.04 |  |
| Aggression | 93 | .80 (.77) | 0-2 | 0.36 |  | -1.22 |  |
| Aggression | 94 | .43 (.65) | 0-2 | 1.22 |  | 0.28 |  |
| Aggression | 95 | .73 (.71) | 0-2 | 0.44 |  | -0.97 |  |
| Aggression | 97 | .12 (.37) | 0-2 | 3.26 |  | 10.55 |  |
| Aggression | 104 | .55 (.67) | 0-2 | 0.84 |  | -0.46 |  |
| Scale | Item | M (SD) | Range | Skew |  | Kurtosis |  |
| Attention | 1 | .35 (.59) | 0-2 | 1.5 |  | 1.17 |  |
| Attention | 8 | .63 (.70) | 0-2 | 0.65 |  | -0.76 |  |
| Attention | 10 | .94 (.78) | 0-2 | 0.1 |  | -1.36 |  |
| Attention | 13 | .12 (.36) | 0-2 | 3.07 |  | 9.31 |  |
| Attention | 17 | .24 (.48) | 0-2 | 1.84 |  | 2.57 |  |
| Attention | 41 | .46 (.64) | 0-2 | 1.08 |  | 0.02 |  |
| Attention | 45 | .23 (.51) | 0-2 | 2.15 |  | 3.71 |  |
| Attention | 46 | .09 (.35) | 0-2 | 3.99 |  | 16.07 |  |
| Attention | 62 | .22 (.49) | 0-2 | 2.13 |  | 3.75 |  |
| Attention | 80 | .12 (.36) | 0-2 | 3.03 |  | 9.03 |  |

*Note.* Acceptable skew and kurtosis operationalized as less than 2 and kurtosis of less than 7 (Bryne et al., 2010)

**Table 2**

*Dichotomized Item Frequencies for Externalizing Subscales*

| Scale | Item | Mean (SD) | Range | Did not endorse | Endorsed |
| --- | --- | --- | --- | --- | --- |
| Aggression | 3 | -- | 0-1 | -- | -- |
| Aggression | 7 | .39 (.49) | 0-1 | 681 | 437 |
| Aggression | 16 | .31 (.46) | 0-1 | 774 | 344 |
| Aggression | 19 | -- | -- | -- | -- |
| Aggression | 20 | .34 (.48) | 0-1 | 733 | 385 |
| Aggression | 21 | .32 (.47) | 0-1 | 762 | 356 |
| Aggression | 22 | .59 (.49) | 0-1 | 461 | 657 |
| Aggression | 27 | -- | -- | -- | -- |
| Aggression | 37 | .21 (.41) | 0-1 | 878 | 240 |
| Aggression | 57 | .20 (.40) | 0-1 | 891 | 227 |
| Aggression | 68 | -- | -- | -- | -- |
| Aggression | 74 | -- | -- | -- | -- |
| Aggression | 86 | -- | -- | -- | -- |
| Aggression | 87 | .38 (.49) | 0-1 | 693 | 425 |
| Aggression | 93 | -- | -- | -- | -- |
| Aggression | 94 | .34 (.48) | 0-1 | 734 | 384 |
| Aggression | 95 | -- | -- | -- | -- |
| Aggression | 97 | .1 (.3) | 0-1 | 1003 | 115 |
| Aggression | 104 | -- | -- | -- | -- |
| Scale | Item | Mean (SD) | Range | Did not endorse | Endorsed |
| Attention | 1 | .28 (.45) | 0-1 | 803 | 320 |
| Attention | 8 | -- | -- | -- | -- |
| Attention | 10 | -- | -- | -- | -- |
| Attention | 13 | .11 (.31) | 0-1 | 1002 | 121 |
| Attention | 17 | .22 (.41) | 0-1 | 880 | 243 |
| Attention | 41 | .38 (.48) | 0-1 | 700 | 423 |
| Attention | 45 | .19 (.39) | 0-1 | 909 | 214 |
| Attention | 46 | .07 (.26) | 0-1 | 1089 | 84 |
| Attention | 62 | .19 (.39) | 0-1 | 909 | 214 |
| Attention | 80 | .11 (.31) | 0-1 | 1001 | 122 |

*Note*. The decision about which items to dichotomize was informed by a visual review of the item response distributions. Items with sparse upper distributions (defined as less than 10 percent of the sample) were dichotomized. Ten percent was decided upon as it would result in the smallest cell size still being above approximately 100.

**Figure 1**

*MNLFA Model for the Aggression Subscale*


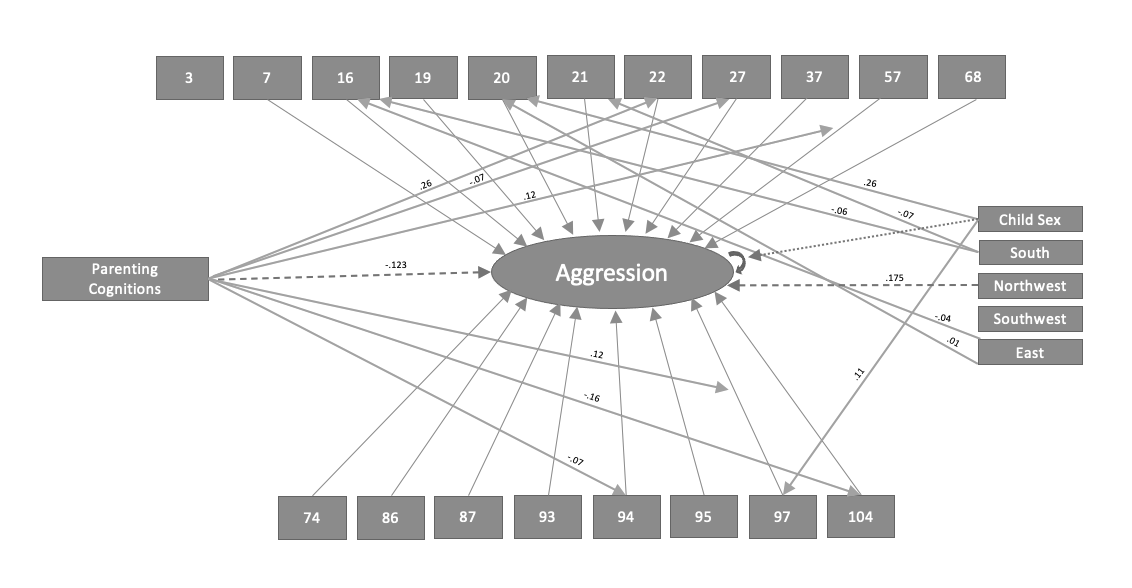


*Note.* Only significant DIF or impact effects are illustrated. Solid lines reflect DIF, dashed lines reflect mean impact, and dotted lines reflect variance impact.

**Figure 2**

*MNLFA Model for the Attention Subscale*


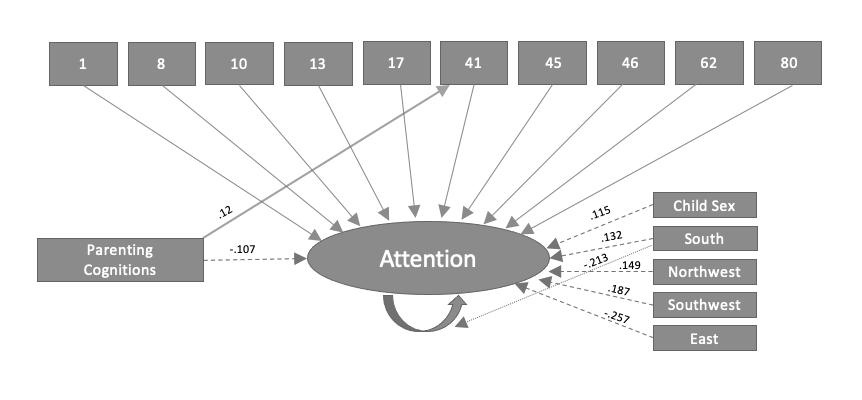


*Note.* Only significant DIF or impact effects are illustrated. Solid lines reflect DIF, dashed lines reflect mean impact, and dotted lines reflect variance impact.
